# Protected-Airway Local/Regional Analgesia-Dominant Strategy Versus General Anesthesia and ICU Length of Stay in Elderly Patients with Traumatic Intracranial Hemorrhage: A Propensity Score-Matched Cohort Study

**DOI:** 10.3390/medicina62071265

**Published:** 2026-06-30

**Authors:** Cheol Lee, Taewan Won

**Affiliations:** 1 Department of Anesthesiology and Pain Medicine, Wonkwang University School of Medicine Hospital, 895 Muwang-ro, Iksan-si 54538, Jeonbuk, Republic of Korea; 2 Institute of Wonkwang Medical Science, Wonkwang University School of Medicine, 895 Muwang-ro, Iksan-si 54538, Jeonbuk, Republic of Korea; 3 Department of Surgery, Wonkwang University School of Medicine Hospital, 895 Muwang-ro, Iksan-si 54538, Jeonbuk, Republic of Korea

**Keywords:** head trauma, chronic subdural hematoma, burr-hole surgery, general anesthesia, protected airway, local/regional analgesia, ICU length of stay

## Abstract

*Background/Objectives*: Older adults undergoing surgery for intracranial hemorrhagic lesions after head trauma are clinically heterogeneous, and burr-hole drainage for trauma-related chronic or localized subdural hematoma differs substantially from craniotomy for acute lesions. We evaluated whether a protected-airway local/regional analgesia-dominant strategy (LA), in which airway protection was maintained but continuous maintenance-dose general anesthesia was not planned, was associated with shorter intensive care unit (ICU) stay than conventional general anesthesia (GA). *Materials and Methods*: In this single-center propensity score-matched retrospective cohort study, 330 patients aged ≥65 years with admission Glasgow Coma Scale (GCS) ≤ 8 who underwent surgery between 2015 and 2024 were analyzed. The LA approach was a pragmatic, jointly selected anesthesiologist–neurosurgeon strategy for carefully selected short burr-hole or localized subdural hematoma procedures; it was not an awake technique and not a protocol of leaving an intubated patient without drugs for airway-device tolerance. A protected airway could include a tracheal tube, supraglottic airway, or preexisting endotracheal tube according to clinical context, and titrated analgesic, sedative, or rescue anesthetic medications were permitted when clinically required. Propensity scores were estimated using age, sex, admission GCS, American Society of Anesthesiologists class, and Charlson Comorbidity Index; lesion category, procedure type, antithrombotic therapy, and intraoperative hypotension were examined as major sources of residual confounding. *Results*: After matching, the LA group had shorter ICU stay (4 [IQR 2–6] vs. 6 [4–10] days; *p* < 0.001). Negative binomial regression showed a 28% lower expected ICU stay with LA (incidence rate ratio 0.72, 95% CI 0.58–0.89; *p* = 0.003), and competing-risk analysis showed faster alive ICU discharge (subdistribution hazard ratio 1.41, 95% CI 1.08–1.84; *p* = 0.012). *Conclusions*: In this heterogeneous retrospective cohort, the LA strategy was associated with shorter ICU stay, particularly within selected burr-hole-dominant cases. These findings are hypothesis-generating and should not be interpreted as proof of superiority across acute traumatic brain injury, all lesion types, or all neurosurgical procedures.

## 1. Introduction

Older adults presenting after head trauma frequently have surgically treated intracranial hemorrhagic lesions ranging from acute subdural hematoma (ASDH) or epidural hematoma (EDH) to delayed, trauma-related chronic subdural hematoma (cSDH) [1,2,3,4,5]. Although a low admission Glasgow Coma Scale (GCS) score identifies high clinical acuity, this population is not equivalent to a homogeneous diffuse severe traumatic brain injury phenotype, because focal mass lesions and delayed cSDH are common in older adults after low-energy trauma [1,2,3,4,5].

For craniotomy or craniectomy, general anesthesia (GA) remains the standard anesthetic approach. In contrast, selected burr-hole or localized subdural hematoma procedures may be managed with scalp block and/or local infiltration while airway protection is maintained because of depressed consciousness, aspiration risk, ventilatory need, or the need for immobility [6,7,8,9,10,11,12,13]. This should not be confused with textbook awake neurosurgery or with leaving an intubated patient untreated: airway-device tolerance, ventilator synchrony, movement control, and surgical conditions require continuous clinical assessment and titrated analgesic, sedative, neuromuscular-blocking, or rescue hypnotic medications when necessary. Because this protected-airway local/regional analgesia-dominant strategy is not standardized and has limited direct support in PubMed-indexed literature or textbooks, its description, indications, and limitations require explicit clarification before outcome comparisons can be interpreted.

Prior literature has addressed both clinical outcomes and perioperative considerations in this population. Severe traumatic brain injury (TBI) cohort meta-analyses have documented substantial mortality heterogeneity associated with injury severity and physiological reserve [14]. Burr-hole evacuation, including approaches using intracavitary urokinase for acute subdural hematoma and delayed burr-hole evacuation in older adults with low-energy trauma, has been described as a less invasive alternative in selected cases [15,16]. In neuroanesthesia more broadly, outcome determinants extend beyond the choice of maintenance agent to encompass patient selection, intraoperative hemodynamic stability, and adherence to contemporary severe TBI management standards [17,18]. In chronic subdural hematoma drainage specifically, local anesthesia with sedation has been associated with shorter recovery than general anesthesia [19]. However, comparative data that span both acute and delayed hemorrhagic lesions in elderly patients, using rigorous propensity-based adjustment, remain limited.

This propensity score-matched retrospective cohort study aimed to evaluate whether a protected-airway local/regional analgesia-dominant approach, compared with conventional GA, was associated with shorter ICU stay in elderly patients undergoing surgery for intracranial hemorrhagic lesions after head trauma. We prespecified a cautious interpretation, emphasizing that anesthetic selection was linked to lesion category, procedure type, physiological reserve, and surgical judgment, and that prospective randomized validation would be required before any change in practice could be recommended.

## 2. Materials and Methods

### 2.1. Study Design and Population

This propensity score-matched retrospective cohort study was conducted at a Level I trauma center. The study was reported in accordance with the Strengthening the Reporting of Observational Studies in Epidemiology (STROBE) guideline for cohort studies. We identified 330 patients aged ≥65 years with admission GCS ≤ 8 who underwent surgery for intracranial hemorrhagic lesions after documented head trauma or a trauma-related history between January 2015 and December 2024. The cohort included acute subdural hematoma (ASDH), trauma-related cSDH, epidural hematoma (EDH), intraparenchymal hemorrhage (ICH), and traumatic subarachnoid hemorrhage (tSAH). Because delayed cSDH was intentionally retained in the cohort, the study population should be regarded as clinically heterogeneous rather than as a uniform severe TBI cohort.

Eligible patients were those aged 65 years or older with a GCS score of 8 or below at admission, whose intracranial hemorrhagic lesion was confirmed by computed tomography (CT) or magnetic resonance imaging (MRI), who underwent surgery within 72 h of hospital admission or neurosurgical decision-making, and for whom complete anesthesia and ICU records were available. For cSDH, a documented antecedent traumatic event was required, but the inciting trauma itself could have preceded admission by days to weeks. Patients who died within 24 h of admission, those with preexisting neurological or cognitive disorders, and those with incomplete outcome data were excluded.

### 2.2. Ethical Approval

The study was conducted in accordance with the Declaration of Helsinki and approved by the Institutional Review Board of Wonkwang University Hospital (protocol code WKUH 2025-09-004; approved 8 September 2025). Patient consent was waived because this was a retrospective review of de-identified medical records. The study protocol had no role in anesthetic, surgical, or ICU decision-making.

### 2.3. Definition, Rationale, and Clinical Selection of Anesthetic Strategy

Anesthetic strategy was classified as follows. General anesthesia (GA): induction with intravenous agents, airway instrumentation, and continuous maintenance with inhalational anesthetics (sevoflurane or desflurane) or propofol-remifentanil total intravenous anesthesia throughout the procedure. Protected-airway local/regional analgesia-dominant strategy (LA): scalp nerve block (including occipital branches when indicated) and/or local infiltration at the surgical site, with a protected airway maintained during the procedure but without planned continuous maintenance-dose volatile anesthesia or propofol-remifentanil GA. A “protected airway” in this study meant that airway protection and ventilatory support were secured by a tracheal tube, supraglottic airway, or preexisting endotracheal tube, depending on preoperative neurological status, aspiration risk, ventilatory status, and anticipated procedural duration. The LA approach was not an awake technique and was not a protocol of withholding all anesthetic or analgesic medications after airway control. Brief induction agents, neuromuscular blockers for airway management, local/regional analgesia, and titrated rescue opioid, sedative, hypnotic, or neuromuscular-blocking drugs were permitted when needed for airway-device tolerance, ventilator synchrony, patient movement, hemodynamic control, or surgical conditions. Cases requiring conversion to continuous GA maintenance were recorded and retained in the protected-airway LA-dominant group for the primary strategy-assigned analysis, because conversion occurred after the clinical selection and initiation of the LA strategy and represents a clinically relevant event of that strategy rather than a pre-treatment baseline characteristic. Selection of LA versus GA was made collaboratively by the attending anesthesiologist and neurosurgeon after considering lesion pattern, anticipated surgical duration, need for craniotomy versus burr-hole drainage, airway and aspiration risk, intracranial pressure concern, and hemodynamic stability. LA was generally considered only for selected burr-hole drainage or localized subdural hematoma cases in which surgical stimulation and expected duration were limited. Detailed dose-level distributions of volatile versus TIVA maintenance, airway device type, block combinations, local-anesthetic volume, rescue opioid/sedative administration, and neuromuscular blocker use were not consistently extractable from the retrospective record and are therefore not compared quantitatively.

### 2.4. Data Collection

Data were extracted from electronic medical records and encompassed the following: demographic characteristics (age, sex); injury and clinical characteristics including GCS at admission, primary lesion type (acute subdural hematoma [ASDH], trauma-related chronic subdural hematoma [cSDH], epidural hematoma [EDH], intraparenchymal hemorrhage [ICH], traumatic subarachnoid hemorrhage [tSAH], or other), lesion chronicity (acute vs. chronic), imaging severity markers (midline shift > 5 mm, basal cistern effacement, multi-compartment involvement), anticoagulant or antiplatelet use at admission, including dual antiplatelet therapy when documented, ASA physical status class, and Charlson Comorbidity Index (CCI); intraoperative variables including anesthesia type, airway device when available, surgery type (craniotomy/craniectomy or burr-hole), surgical duration, and the presence of intraoperative hypotension (defined as mean arterial pressure < 65 mmHg for >5 min); and postoperative outcomes. Drug-specific antithrombotic dose, indication, exact timing of last administration, reversal strategy, and coagulation–normalization data were incompletely available and were therefore considered an important limitation rather than a fully adjusted covariate set.

### 2.5. Outcome Measures

The primary outcome was the length of ICU stay (measured in days). Secondary outcomes included in-hospital mortality, duration of mechanical ventilation (days), incidence of hospital-acquired pneumonia (HAP), acute respiratory distress syndrome (ARDS), sepsis or systemic inflammatory response syndrome (SIRS), postoperative delirium, reintubation within 48 h of extubation, ICU readmission during the same hospitalization, total hospital length of stay (days), and discharge disposition (home, rehabilitation facility, long-term care hospital, hospice, or death). All outcomes were retrieved from the electronic medical records using standardized definitions aligned with institutional and international guidelines.

### 2.6. Propensity Score Matching

To reduce selection bias, propensity scores were estimated using a logistic regression model with anesthetic strategy (GA vs. LA) as the dependent variable and the following covariates: age, sex, admission GCS, ASA class, and CCI. One-to-one nearest-neighbor matching without replacement was performed using a caliper of 0.2 standard deviations of the logit of the propensity score [20]. Patients outside the region of common support were excluded (*n* = 5 from the GA group). This yielded 100 matched pairs (total *n* = 200). Propensity matching was used to improve measured baseline comparability, but it cannot make a retrospective nonrandomized cohort equivalent to a randomized trial.

The primary propensity model used baseline variables that were consistently available before definitive anesthetic selection: age, sex, admission GCS, ASA class, and CCI. Variable selection followed established recommendations that favor covariates plausibly related to both treatment assignment and the outcome while avoiding instruments and downstream mediators [21,22]. Lesion category, lesion chronicity, surgery type, and intraoperative hypotension were treated as major sources of case-mix heterogeneity and residual confounding and were therefore examined in subgroup, sequential adjustment, and descriptive sensitivity analyses rather than interpreted as fully exchangeable across matched pairs. This choice reflects a methodological limitation of retrospective comparative effectiveness analysis rather than evidence of causal equivalence between treatment groups.

Covariate balance was assessed using standardized mean differences (SMD), with a threshold of <0.10 indicating adequate balance. SMD values are reported in Table 1 for both pre-match and post-match comparisons. Detailed post-match baseline characteristics for the primary propensity covariates are provided in Supplementary Table S1, and a summary of covariate balance before and after matching is provided in Supplementary Table S1.

### 2.7. Statistical Analysis

Continuous variables were reported as means ± standard deviations or medians [interquartile ranges] according to distribution and were compared using independent *t*-tests or Mann–Whitney U tests. Categorical variables were presented as counts (percentages) and analyzed with chi-square tests. All tests were two-sided, and statistical significance was defined as *p* < 0.05. Analyses were performed both before and after PSM for baseline characteristics and outcomes.

For the primary outcome, ICU length of stay was analyzed using three complementary approaches: (1) Mann–Whitney U test for median comparison; (2) negative binomial regression for count-based modeling, yielding an incidence rate ratio (IRR); and (3) Fine–Gray subdistribution hazard model treating alive ICU discharge as the event of interest and in-hospital death as the competing event, yielding a subdistribution hazard ratio (SHR). This competing-risk framework addresses the potential for informative censoring by death, which could bias simple length-of-stay comparisons [23].

Multivariable logistic regression identified independent predictors of prolonged ICU stay (defined as above the overall matched-cohort median) in both unmatched and matched cohorts, adjusting for variables significant in univariate analyses. Because dichotomization of ICU stay is clinically intuitive but statistically reductive, these logistic models were considered supportive rather than primary analyses. Sensitivity analyses additionally adjusted for surgery type and intraoperative hypotension to quantify residual confounding. Because inclusion of trauma-related cSDH intentionally increased heterogeneity, a post hoc descriptive sensitivity analysis excluding cSDH was also performed to evaluate whether the direction of association persisted in a more acute-lesion cohort; this analysis was considered exploratory because of reduced sample size and persistent procedural imbalance. The E-value was calculated to contextualize, but not exclude, the potential impact of unmeasured confounding [24]. Secondary outcomes were interpreted as exploratory, and no formal multiplicity adjustment was applied. Patients who were initially managed with the LA-dominant strategy but subsequently required conversion to continuous GA maintenance were retained in the LA-dominant group for the primary strategy-assigned analysis. These conversions occurred after the LA strategy had been clinically selected and initiated; therefore, they were considered post-baseline escalation events within the LA pathway rather than baseline GA cases. A post hoc exclusion sensitivity analysis of these four patients was not performed because such exclusion could introduce post-baseline selection bias by removing clinically relevant failures or escalations of the LA pathway and could make LA-dominant management appear more favorable. In addition, because propensity score matching was performed according to the initial anesthetic strategy, removing these patients after matching would alter the matched analytic cohort and would require reassessment of covariate balance and re-estimation of outcome models; therefore, simple post hoc exclusion of these cases was considered methodologically inconsistent with the primary matched design. The frequency and reasons for conversion are reported explicitly in Section 3.

## 3. Results

### 3.1. Baseline Characteristics Before and After Propensity Score Matching

Before matching, patients receiving GA (*n* = 230) had greater illness severity and perioperative risk than those receiving LA (*n* = 100). GA patients presented with lower GCS scores (median 5 [IQR 3–6] vs. 7 [5–8]; *p* < 0.001), a higher proportion of ASA class ≥ III (61% vs. 50%; *p* = 0.066), and higher CCI (4.3 ± 1.7 vs. 3.6 ± 1.7; *p* < 0.001). Surgical duration was longer (168 ± 55 vs. 140 ± 40 min; *p* < 0.001), and intraoperative hypotension was more frequent (46% vs. 20%; *p* < 0.001). Surgery type distributions also differed, with burr-hole procedures overrepresented in LA (Table 1).

After PSM, 100 GA and 100 LA patients were balanced on the primary baseline covariates used in the propensity model (age, sex, admission GCS, ASA class, CCI; all SMD < 0.10). However, residual differences persisted in surgical duration (160 ± 50 vs. 140 ± 40 min; *p* = 0.002), intraoperative hypotension (40% vs. 20%; *p* = 0.003; SMD = 0.45), and surgery type (craniotomy 65% vs. 50%; *p* = 0.040; SMD = 0.31). These imbalances are clinically important because operative invasiveness and hemodynamic instability plausibly affect ICU stay independently of anesthetic strategy. Accordingly, the matched analysis should be interpreted as an overall association within a heterogeneous cohort, not as a clean comparison of interchangeable anesthetic techniques.

Among the full cohort, predominant primary lesions were trauma-related cSDH (86, 26.1%), ASDH (58, 17.6%), EDH (87, 26.4%), intraparenchymal hemorrhage (66, 20.0%), and tSAH (33, 10.0%). Multi-compartment involvement occurred in 92 (27.9%). Midline shift > 5 mm and basal cistern effacement were present in 132 (40.0%) and 116 (35.2%), respectively. Anticoagulant or antiplatelet use at admission was documented in 78 (23.6%), including dual antiplatelet therapy when specifically recorded. Procedures comprised burr-hole decompression/drainage in 115 (34.8%) and craniotomy/craniectomy in 215 (65.2%). In Table 1, SDH is reported as a combined category for compact presentation; this combined category includes both ASDH and trauma-related cSDH, and chronicity-specific interpretation is addressed in the sensitivity analyses and Discussion.

In the LA-intended subgroup (*n* = 100), conversion to continuous GA maintenance occurred in 4 (4.0%) cases due to refractory agitation (*n* = 2), intraoperative brain swelling (*n* = 1), and respiratory compromise (*n* = 1). These patients were retained in the LA-dominant group for the primary strategy-assigned analysis, consistent with the preoperative/intraoperative clinical strategy initially selected. No post hoc exclusion sensitivity analysis was performed because conversion occurred after initiation of the LA-based strategy and exclusion of these cases could remove clinically relevant escalation events from the LA pathway and disturb the propensity score-matched analytic cohort.

### 3.2. Clinical Outcomes Before and After Matching

Before matching, LA was associated with shorter ICU length of stay (4 [2–6] vs. 7 [4–11] days; *p* < 0.001) and lower in-hospital mortality (13% vs. 33%; *p* < 0.001). LA patients also had fewer ventilator days and lower incidence of hospital-acquired pneumonia, postoperative delirium, ARDS, and sepsis/SIRS (all *p* ≤ 0.002); however, these crude comparisons reflect marked baseline and procedural differences between groups.

In the matched cohort, ICU stay remained shorter in the LA group (4 [2–6] vs. 6 [4–10] days; *p* < 0.001). Negative binomial regression estimated a 28% lower expected ICU stay with LA (IRR 0.72, 95% CI 0.58–0.89; *p* = 0.003), and Fine–Gray competing-risk analysis showed faster alive ICU discharge (SHR 1.41, 95% CI 1.08–1.84; *p* = 0.012). These results should be interpreted as adjusted associations within the matched cohort. They do not establish that LA itself caused shorter ICU stay across all lesion categories or procedures, because residual differences in surgery type, hypotension, lesion chronicity, and unmeasured intraoperative management remained.

In-hospital mortality was lower in the LA group (13% vs. 29%; *p* = 0.006). Ventilator days were reduced (2 [1–4] vs. 4 [2–7] days; *p* = 0.010). LA was also associated with lower incidence of hospital-acquired pneumonia (15% vs. 31%; *p* = 0.007), postoperative delirium (13% vs. 25%; *p* = 0.030), ARDS (14% vs. 26%; *p* = 0.030), and sepsis/SIRS (18% vs. 32%; *p* = 0.020). Reintubation within 48 h (6% vs. 14%; *p* = 0.060) and ICU readmission (8% vs. 16%; *p* = 0.080) numerically favored LA without meeting the prespecified significance threshold.

A post hoc analysis restricted to ICU survivors (excluding all patients who died during the ICU stay, not only those within 24 h of admission) confirmed that ICU length of stay remained shorter in the LA group (median 4 [2–6] vs. 5 [3–9] days; *p* = 0.008), indicating that the observed difference was not driven by differential early mortality (Table 2).

**Table 2:** Comparison of clinical outcomes according to anesthetic strategy.

	Before Matching			After Matching		
Outcome	GA (*n* = 230)	LA (*n* = 100)	*p*	GA (*n* = 100)	LA (*n* = 100)	*p*
Length of ICU Stay (days)	7 [4–11]	4 [2–6]	<0.001	6 [4–10]	4 [2–6]	<0.001
In-Hospital Mortality	76 (33%)	13 (13%)	<0.001	29 (29%)	13 (13%)	0.006
Ventilation Duration (days)	5 [3–8]	2 [1–4]	<0.001	4 [2–7]	2 [1–4]	0.010
Hospital-Acquired Pneumonia	83 (36%)	15 (15%)	<0.001	31 (31%)	15 (15%)	0.007
Postoperative Delirium	67 (29%)	13 (13%)	0.002	25 (25%)	13 (13%)	0.030
ARDS	71 (31%)	14 (14%)	0.001	26 (26%)	14 (14%)	0.030
Sepsis or SIRS	83 (36%)	18 (18%)	0.001	32 (32%)	18 (18%)	0.020
Reintubation within 48 h	37 (16%)	6 (6%)	0.012	14 (14%)	6 (6%)	0.060
ICU Readmission	41 (18%)	8 (8%)	0.021	16 (16%)	8 (8%)	0.080
Total Hospital Stay (days)	15 [11–22]	10 [7–15]	<0.001	14 [10–20]	10 [7–15]	0.002
Discharge			<0.001			0.040
Home	51 (22%)	35 (35%)		25 (25%)	35 (35%)	
Rehabilitation Facility	60 (26%)	40 (40%)		30 (30%)	40 (40%)	
Long-Term Care Hospital	32 (14%)	8 (8%)		12 (12%)	8 (8%)	
Hospice	11 (5%)	4 (4%)		4 (4%)	4 (4%)	
Death	76 (33%)	13 (13%)		29 (29%)	13 (13%)	

Data are presented as median [IQR] or *n* (%). Footnote: *p*-values were calculated using Mann–Whitney U test for continuous variables and chi-square test for categorical variables; all tests were two-sided and interpreted with a significance threshold of *p* < 0.05. Secondary outcomes were exploratory and were not adjusted for multiplicity. Abbreviation: GA, general anesthesia; LA, local/regional anesthesia; ICU, intensive care unit; IQR, interquartile range; ARDS, acute respiratory distress syndrome; SIRS, systemic inflammatory response syndrome.

### 3.3. Predictors of Prolonged ICU Stay

In multivariable logistic regression, GA independently predicted prolonged ICU stay before matching (adjusted OR 2.10, 95% CI 1.37–3.21, *p* = 0.001) and after matching (adjusted OR 1.89, 95% CI 1.23–2.91, *p* = 0.004). Admission GCS ≤ 6 was also associated with increased risk (adjusted OR 1.85 pre-match and 1.65 post-match; *p* = 0.003 and 0.020). Sepsis was the strongest predictor (adjusted OR 2.55 pre-match and 2.34 post-match; both *p* < 0.001). ASA class ≥ III was a borderline predictor before matching (adjusted OR 1.62, *p* = 0.030) but not significant post-match (adjusted OR 1.44, *p* = 0.080) (Table 3).

In a sensitivity analysis additionally adjusting for surgery type and intraoperative hypotension, the association between GA and prolonged ICU stay was attenuated but remained statistically significant (adjusted OR 1.62, 95% CI 1.03–2.55; *p* = 0.038), suggesting that procedure type and intraoperative instability account for part, but not all, of the observed association (Supplementary Table S3). The E-value for the primary GA–ICU stay association (OR 1.89) was 3.19. This finding suggests moderate robustness to unmeasured confounding, but it does not rule out clinically plausible residual confounding related to case selection, procedural complexity, or unmeasured intraoperative management.

### 3.4. Sensitivity Analyses and Exploratory Subgroups

In a subset restricted to burr-hole procedures, LA retained an association with shorter ICU stay (median 4 vs. 5 days; adjusted β −0.9 days, 95% CI −1.6 to −0.2; *p* = 0.010). Results were directionally consistent when additionally adjusting for imaging severity markers (midline shift, cistern effacement) and extracranial injury (Abbreviated Injury Scale ≥ 3). In the post hoc descriptive analysis excluding trauma-related cSDH, the direction of the association for ICU stay remained favorable to LA, but the sample size was smaller and residual procedure-type imbalance persisted; therefore, this analysis was considered exploratory and insufficient to support a conclusion for acute traumatic brain injury alone.

A negative-control outcome analysis using surgical wound dehiscence—a complication with no plausible causal link to anesthetic strategy—showed no association between LA and GA (*p* = 0.720), supporting the specificity of the observed associations for the primary and secondary outcomes (Supplementary Table S4).

No significant interaction was observed between anesthetic strategy and primary lesion category (*p* = 0.410) or age ≥ 80 years (*p* = 0.330). Point estimates consistently favored LA for ICU stay across strata, but precision was limited in the EDH/ASDH subgroup requiring craniotomy (Supplementary Table S2).

## 4. Discussion

This propensity score-matched retrospective cohort study found that a protected-airway local/regional analgesia-dominant strategy (LA) was associated with shorter ICU stay than conventional GA in older adults undergoing surgery for intracranial hemorrhagic lesions after head trauma. After matching on the primary baseline covariates, median ICU stay remained two days shorter with LA, and the direction of association was consistent across count-based and competing-risk analyses. However, the cohort was clinically heterogeneous, burr-hole procedures were overrepresented in LA, and residual imbalances in surgery type and intraoperative hypotension remained. These data should therefore be interpreted as hypothesis-generating associations rather than evidence that LA is superior to GA for acute traumatic brain injury or for all neurosurgical procedures.

The present study also clarifies what was meant by “protected-airway” LA. This was not awake neurosurgery and not an intentional sedation hold after endotracheal intubation. In selected cases, scalp block and/or local infiltration were used as the primary antinociceptive technique, while airway protection was maintained with a tracheal tube, supraglottic airway, or preexisting endotracheal tube according to the clinical condition. Continuous maintenance-dose volatile anesthesia or propofol-remifentanil GA was not planned, but titrated opioid, sedative, hypnotic, or neuromuscular-blocking medications could be administered for airway-device tolerance, ventilator synchrony, movement, hemodynamic control, or surgical conditions. This pragmatic strategy is not a universally transferable textbook anesthetic technique; its feasibility depends on careful patient selection, procedural brevity, secure neurosurgical access, and close anesthesiologist–neurosurgeon collaboration.

The rationale for considering LA in selected burr-hole-dominant cases was to reduce exposure to maintenance-dose hypnotic anesthetics, minimize anesthetic-associated hypotension, and potentially shorten postoperative ventilatory and ICU requirements. Nevertheless, the apparent advantage of LA cannot be confirmed mechanistically in this dataset. More favorable case selection is likely: LA was predominantly reserved for trauma-related cSDH or localized SDH amenable to burr-hole drainage, whereas craniotomy/craniectomy and intraoperative hypotension were more common in the GA group. Therefore, even after PSM and sequential adjustment, the observed association may partly reflect procedural invasiveness, lesion biology, baseline neurological status, or unmeasured anesthetic and ICU management rather than the anesthetic strategy itself.

An additional clinically relevant message is that not all lesions in this cohort should be described as classical severe TBI. Older patients with trauma-related cSDH may present days to weeks after the inciting event and differ substantially from patients with acute EDH, ASDH, or diffuse injury. cSDH was retained because it represents a common trauma-related surgical pathway in elderly patients and because the clinical question arose from real-world triage between burr-hole and more invasive procedures. However, its inclusion increases heterogeneity and limits inference for acute traumatic brain injury. Accordingly, the revised manuscript separates ASDH and trauma-related cSDH descriptively, reports SDH grouping transparently in Table 1, and treats the cSDH-excluded analysis as exploratory rather than definitive.

Our findings are broadly consistent with prior work in cSDH and burr-hole populations, where local/regional approaches with light adjunctive sedation have been associated with shorter recovery and fewer complications than GA [11,12,13,19]. However, direct generalization to all elderly neurotrauma patients would be inappropriate, especially for acute craniotomy cases and lesion categories that remained imbalanced after matching. Future studies may also refine selection for minimally invasive neurosurgical drainage by incorporating quantitative imaging, three-dimensional reconstruction, and artificial intelligence (AI)-assisted surgical planning. Similar AI-based concepts are emerging in minimally invasive and oncologic surgery, including diagnostic support, intraoperative guidance, and perioperative risk prediction [25]. These tools were not evaluated in the present cohort, and their mention should be understood as a future research direction rather than an explanation for the observed association.

This study has some limitations. First, the retrospective single-center design remains vulnerable to selection bias and residual confounding despite matching; a prospective randomized or, at minimum, protocolized prospective cohort study would be more appropriate to test this clinical strategy. Second, lesion category, lesion chronicity, surgery type, and intraoperative hypotension were still imbalanced after matching, and the treatment effect attenuated in sequentially adjusted models. Third, detailed preoperative ICU length of stay, preoperative ventilatory status, airway device type, volatile-versus-TIVA distribution, block combinations, local-anesthetic dose, rescue opioid/sedative dose, neuromuscular blocker use, and airway-device tolerance management were not sufficiently complete in the electronic record to permit reliable comparative tabulation. Fourth, older patients frequently receive anticoagulant, antiplatelet, or dual antiplatelet therapy; although antithrombotic exposure at admission was recorded, drug-specific intensity, timing of last dose, reversal strategy, and residual anticoagulant effect were incompletely captured and may confound both surgical timing and outcomes. Fifth, secondary outcomes were exploratory and no multiplicity adjustment was applied. Sixth, long-term functional outcomes were unavailable. Seventh, four LA-intended patients required conversion to continuous GA maintenance. These patients were retained in the primary strategy-assigned analysis to avoid post-baseline exclusion bias because conversion represented a clinically relevant escalation after initiation of the LA-based pathway rather than baseline assignment to GA. A post hoc exclusion sensitivity analysis was not performed because excluding these cases would alter the propensity score-matched analytic cohort and would require reassessment of covariate balance and outcome estimates. Nevertheless, their occurrence underscores the need for prospective protocols with predefined conversion criteria and prespecified per-protocol or as-treated sensitivity analyses.

Taken together, these limitations justify a conservative interpretation. The present analysis supports further prospective study of a protected-airway local/regional analgesia-dominant approach only in carefully selected burr-hole-dominant cases, with standardized documentation of airway device, sedation/analgesia dosing, antithrombotic management, imaging severity, intraoperative hemodynamics, and postoperative ICU protocols. It should not be read as proving superiority over GA across all intracranial hemorrhagic lesions or all neurosurgical procedures.

## 5. Conclusions

In this heterogeneous retrospective cohort of older adults undergoing surgery for intracranial hemorrhagic lesions after head trauma, a protected-airway local/regional analgesia-dominant strategy was associated with shorter ICU stay and fewer in-hospital complications than conventional GA. The association appeared strongest in selected burr-hole-dominant cases, particularly trauma-related cSDH or localized SDH. Because treatment selection was closely linked to lesion type, procedure, hemodynamic risk, and clinician judgment, these findings remain hypothesis-generating and warrant prospective validation before broad practice recommendations are made.

## Figures and Tables

**Table 1:** Baseline characteristics before and after propensity score matching.

	**Before Matching**				**After Matching**			
**Variable**	**GA (** ***n*** ** = 230)**	**LA (** ***n*** ** = 100)**	***p***	**SMD**	**GA (** ***n*** ** = 100)**	**LA (** ***n*** ** = 100)**	***p***	**SMD**
Age (years)	77.1 ± 7.3	76.6 ± 6.2	0.525	0.07	75.8 ± 6.8	76.6 ± 6.2	0.410	0.06
Male sex	156 (68%)	62 (62%)	0.300	0.12	66 (66%)	62 (62%)	0.580	0.08
GCS at admission	5 [3–6]	7 [5–8]	<0.001	0.85	5 [3–7]	7 [5–8]	0.120	0.09
ASA ≥ III	140 (61%)	50 (50%)	0.066	0.22	52 (52%)	50 (50%)	0.890	0.04
Charlson Comorbidity Index	4.3 ± 1.7	3.6 ± 1.7	<0.001	0.41	4.0 ± 1.5	3.6 ± 1.7	0.079	0.07
Surgical Duration (minutes)	168 ± 55	140 ± 40	<0.001	0.56	160 ± 50	140 ± 40	0.002	0.44
Hypotension	106 (46%)	20 (20%)	<0.001	0.57	40 (40%)	20 (20%)	0.003	0.45
Primary lesion category *			0.800				0.800	
SDH (ASDH + trauma-related cSDH)	104 (45%)	40 (40%)			45 (45%)	40 (40%)		
EDH	57 (25%)	30 (30%)			25 (25%)	30 (30%)		
ICH	46 (20%)	20 (20%)			20 (20%)	20 (20%)		
Other (tSAH/mixed)	23 (10%)	10 (10%)			10 (10%)	10 (10%)		
Surgery type			<0.001	0.46			0.040	0.31
Craniotomy	165 (72%)	50 (50%)			65 (65%)	50 (50%)		
Burr hole	65 (28%)	50 (50%)			35 (35%)	50 (50%)		

Data are presented as mean ± SD, median [IQR], or *n* (%). Footnote: *p*-values were calculated using independent *t*-test or Mann–Whitney U test for continuous variables and chi-square test for categorical variables; all tests were two-sided and interpreted with a significance threshold of *p* < 0.05. SMD, standardized mean difference. SMD < 0.10 indicates adequate balance for the covariates included in the primary propensity model. * Primary lesion category: SDH includes ASDH and trauma-related cSDH; subgroup analysis by lesion category and surgery type is provided in Supplementary Table S2.

**Table 3:** Multivariable regression analysis for predictors of prolonged ICU stay.

Variable	Before Matching			After Matching		
	Adj. OR	95% CI	*p*	Adj. OR	95% CI	*p*
General anesthesia	2.10	1.37–3.21	0.001	1.89	1.23–2.91	0.004
GCS ≤ 6	1.85	1.23–2.78	0.003	1.65	1.10–2.50	0.020
Sepsis	2.55	1.58–4.12	<0.001	2.34	1.45–3.77	<0.001
ASA class ≥ III	1.62	1.06–2.47	0.030	1.44	0.95–2.18	0.080

Footnote: Multivariable logistic regression was used with prolonged ICU stay (defined as above the median) as the dependent variable. Variables included in the model were those significant in univariate analysis. These models are supportive analyses and do not eliminate residual confounding related to lesion category, procedure type, or detailed intraoperative management. Abbreviation: OR, odds ratio; CI, confidence interval; GCS, Glasgow Coma Scale; ICU, intensive care unit; ASA, American Society of Anesthesiologists.

## Data Availability

The datasets presented in this article are not readily available because the data contain sensitive patient information and are subject to institutional data governance policies. Requests to access the datasets should be directed to ironyii@wku.ac.kr.

## Supplementary Materials

The following supporting information can be downloaded at https://www.mdpi.com/article/10.3390/medicina62071265/s1, Table S1. Baseline characteristics and covariate balance assessment before and after propensity score matching; Table S2. Subgroup Analysis of ICU Length of Stay by Injury Type and Surgery Type; Table S3. Sensitivity Analysis: Association of General Anesthesia with Prolonged ICU Stay Under Sequentially Adjusted Models; Table S4. Negative-Control Outcome Analysis (Surgical Wound Dehiscence).

## Abbreviations

The following abbreviations are used in this manuscript:

TBI	Traumatic brain injury
GCS	Glasgow Coma Scale
ICU	Intensive care unit
GA	General anesthesia
LA	Protected-airway local/regional analgesia-dominant strategy
PSM	Propensity score matching
SDH	Subdural hematoma
ASDH	Acute subdural hematoma
cSDH	Chronic subdural hematoma
EDH	Epidural hematoma
ICH	Intracerebral hemorrhage
tSAH	Traumatic subarachnoid hemorrhage
ASA	American Society of Anesthesiologists
CCI	Charlson Comorbidity Index
LOS	Length of stay
HAP	Hospital-acquired pneumonia
ARDS	Acute respiratory distress syndrome
SIRS	Systemic inflammatory response syndrome
SMD	Standardized mean difference
IRR	Incidence rate ratio
SHR	Subdistribution hazard ratio
OR	Odds ratio
CI	Confidence interval
IQR	Interquartile range
CT	Computed tomography
MRI	Magnetic resonance imaging
EMR	Electronic medical records
AIS	Abbreviated Injury Scale
