# Supplementary material for: Protected-Airway Local/Regional Analgesia-Dominant Strategy Versus General Anesthesia and ICU Length of Stay in Elderly Patients with Traumatic Intracranial Hemorrhage: A Propensity Score-Matched Cohort Study
Source: Medicina (Kaunas). 2026 Jun 30;62(7):1265. doi: 10.3390/medicina62071265 (PMC13414364; doi:10.3390/medicina62071265)
Supplement: Supplementary file 1 [file medicina-62-01265-s001.zip › medicina-4318248-Supplementary Materials.pdf]

Supplementary Materials

Protected-Airway Local/Regional Analgesia-Dominant Strategy Versus General Anesthesia and ICU Length of Stay in Elderly Patients with Traumatic Intracranial Hemorrhage: A Propensity Score-Matched Cohort Study

Cheol Lee and Taewan Won

Supplementary Note. The cohort intentionally included both acute hemorrhagic lesions and trauma-related chronic subdural hematoma. For delayed lesions such as chronic subdural hematoma, the eligibility window referred to surgery within 72 h of hospital admission or neurosurgical decision-making, not within 72 h of the remote inciting trauma. Detailed distributions of induction drugs, volatile versus TIVA maintenance, block combinations, rescue opioid administration, local-anesthetic dose, preoperative ventilatory status, and preoperative ICU stay were not consistently extractable from the retrospective record and therefore could not be tabulated reliably.

Table S1. Baseline characteristics and covariate balance assessment before and after propensity score matching.

Post-matching baseline characteristics			
**Variable**	**GA (*n* = 100)**	**LA (*n* = 100)**	**SMD after matching**
Age (years), mean ± SD	75.8 ± 6.8	76.6 ± 6.2	0.060
Male sex, n (%)	66 (66%)	62 (62%)	0.080
GCS at admission, median [IQR]	5 [3–7]	7 [5–8]	0.090
ASA class ≥III, n (%)	52 (52%)	50 (50%)	0.040
CCI, mean ± SD	4.0 ± 1.5	3.6 ± 1.7	0.070
**Covariate balance before and after matching**			
**Covariate**	**SMD before matching**	**SMD after matching**	**Balance achieved**
Age	0.07	0.060	Yes
Sex	0.12	0.080	Yes
GCS at admission	0.85	0.090	Yes
ASA class ≥III	0.22	0.040	Yes
CCI	0.41	0.070	Yes

Data are presented as mean ± SD, median [IQR], or n (%). SMD, standardized mean difference. Balance achieved indicates SMD < 0.10 after matching for the primary baseline covariates. GA, general anesthesia; LA, protected-airway local/regional analgesia-dominant strategy; GCS, Glasgow Coma Scale; ASA, American Society of Anesthesiologists; CCI, Charlson Comorbidity Index.

Table S2. Exploratory subgroup and sensitivity summary for ICU length of stay.

Analysis	Population	Known sample/stratum	Result	p-value	Interpretation
Burr-hole procedure subset	Matched cohort restricted to burr-hole procedures	GA n = 35; LA n = 50	Adjusted β −0.9 days (95% CI −1.6 to −0.2)	0.010	Favored LA; exploratory because procedure restriction does not remove all residual confounding.
Primary lesion category interaction	Matched cohort	Overall interaction test	No statistically significant interaction	0.410	No evidence of differential association by lesion category; precision was limited in smaller strata.
Age ≥80 years interaction	Matched cohort	Overall interaction test	No statistically significant interaction	0.330	No evidence of differential association by age ≥80 years.
Excluding trauma-related cSDH	Post hoc descriptive sensitivity analysis	Acute-lesion subset	Direction of association remained favorable to LA	Not used for definitive inference	Exploratory because sample size was reduced and procedure-type imbalance persisted.

These exploratory analyses summarize procedure-restricted, interaction, and post hoc sensitivity findings. They are intended to show residual heterogeneity and should not be interpreted as proving exchangeability across all matched pairs or causal superiority of anesthetic strategy. GA, general anesthesia; LA, protected-airway local/regional analgesia-dominant strategy; ICU, intensive care unit; LOS, length of stay; CI, confidence interval; cSDH, chronic subdural hematoma.

Table S3. Supportive sensitivity analysis: association of general anesthesia with prolonged ICU stay under sequentially adjusted models.

Model	Adj. OR	95% CI	p-value
Base (age, sex, GCS, ASA, CCI)	1.89	1.23–2.91	0.004
+ Imaging severity (midline shift, cistern)	1.82	1.18–2.83	0.007
+ Extracranial injury (AIS ≥ 3)	1.85	1.19–2.87	0.006
+ Surgery type and intraoperative hypotension	1.62	1.03–2.55	0.038

OR, odds ratio; CI, confidence interval. All models use multivariable logistic regression in the matched cohort (n = 200). These analyses are supportive because residual confounding by lesion pattern, procedure type, and incompletely captured anesthetic detail may remain. GCS, Glasgow Coma Scale; ASA, American Society of Anesthesiologists; CCI, Charlson Comorbidity Index; AIS, Abbreviated Injury Scale.

Table S4. Negative-control outcome analysis: surgical wound dehiscence by anesthetic strategy (matched cohort).

**Outcome**	**GA (*n*=100)**	**LA (*n*=100)**	**p-value**	**OR (95% CI)**
Wound dehiscence	7 (7.0%)	5 (5.0%)	0.720	0.70 (0.21–2.30)

Surgical wound dehiscence was selected as a negative-control outcome because it has no plausible causal link to anesthetic strategy. The absence of a significant association (p = 0.720) supports the specificity of the observed associations, but it does not exclude residual confounding in the primary analyses. OR, odds ratio; CI, confidence interval. GA, general anesthesia; LA, protected-airway local/regional analgesia-dominant strategy.
